# Evaluating the Impact of CYP2D6 Phenotype on Fluvoxamine Pharmacokinetics in Geriatric Patients Using Physiologically Based Pharmacokinetic Modeling

**DOI:** 10.3390/pharmaceutics18020232

**Published:** 2026-02-11

**Authors:** Eunjin Hong

**Affiliations:** College of Pharmacy, Dongguk University-Seoul, 32 Dongguk-Ro, Goyang-si 10326, Republic of Korea; ejhong@dongguk.edu

**Keywords:** pharmacogenetics, pharmacogenomics, geriatrics, CYP2D6 polymorphism, antidepressant, fluvoxamine, physiologically based pharmacokinetic (PBPK), elderly

## Abstract

**Background/Objectives**: Fluvoxamine is commonly prescribed for depressive disorders in elderly patients, a population that frequently exhibits variable drug responses and increased susceptibility to adverse effects due to age-related physiological changes. CYP2D6 polymorphisms may further affect fluvoxamine pharmacokinetics in elderly patients, complicating dose optimization for this group. Previous pharmacogenetic studies examining the impact of CYP2D6 phenotype on fluvoxamine treatment outcomes have primarily focused on younger adults, leaving a gap in understanding its effects on the elderly. **Methods**: The impact of CYP2D6 phenotypes on fluvoxamine exposure in geriatrics was evaluated using a physiologically based pharmacokinetic (PBPK) modeling approach incorporating geriatric-specific physiological parameters. **Results**: The fluvoxamine PBPK model was verified using clinical pharmacokinetic data from younger and older adults, along with phenotype-dependent exposure differences between CYP2D6 poor metabolizers (PMs) and extensive metabolizers (EMs). Simulations showed that steady-state exposure in elderly patients was 1.8-fold higher than those in younger adults, and 2.1-fold higher in CYP2D6 PMs compared with EMs. Based on these simulations, doses of approximately 50 mg/day for PMs, 50–100 mg/day for intermediate metabolizers (IMs), 100 mg/day for EMs, and 150–200 mg/day for ultrarapid metabolizers (UMs) may be appropriate for elderly patients, accompanied by cautious dose escalation and clinical monitoring. **Conclusions**: These findings suggest that CYP2D6 genotype-guided dosing may be a useful strategy for optimizing fluvoxamine therapy in elderly patients, with the potential to improve treatment outcomes while minimizing the risk of adverse drug reactions in this high-risk population.

## 1. Introduction

Depression in older adults is highly prevalent and constitutes a serious health concern [1]. Approximately 15% of the geriatric population have clinically significant depressive symptoms, which significantly diminish their health-related quality of life [2,3,4]. Selective serotonin reuptake inhibitors (SSRIs) are the first-line agents for the management of moderate-to-severe depression in this population. Like other SSRIs, fluvoxamine is an efficacious antidepressant in geriatric depression, and is generally well tolerated largely because it lacks the anticholinergic and anti-adrenergic activities of tricyclic antidepressants [5,6].

Despite its favorable safety profile, elderly patients with depression frequently exhibit heterogeneous therapeutic responses to fluvoxamine, complicating optimal dose selection and titration [7,8]. Age-related physiological changes including reduced hepatic metabolic capacity can increase systemic exposure to fluvoxamine [9,10]. This is particularly relevant given that fluvoxamine is primarily metabolized by CYP2D6, enzymes known to exhibit age-related reductions in activity [11]. Similar age-associated decreases in clearance have been reported for several CYP2D6 substrates, including paroxetine, nortriptyline, and risperidone, which demonstrate increased systemic exposure in geriatrics [12,13,14]. Consistent with these changes, fluvoxamine clearance has been reported to be approximately 50% lower in elderly patients compared with younger adults, resulting in higher plasma concentrations [15]. Elevated exposure may increase the risk of dose-dependent adverse events, including clinically significant hyponatremia [16]. Accordingly, the package insert recommends a lower starting dose with gradual titration in elderly patients, often below the typical adult maintenance dose of 100–300 mg/day [15]. However, detailed clinical guidance regarding fluvoxamine dosing in older adults remains limited. Clinical studies in geriatric populations have reported substantial inter-individual variability in both efficacy and tolerability [8,17]. Collectively, these observations underscore the clinical importance of accounting for age-related pharmacokinetic changes and implementing individualized dosing strategies when prescribing fluvoxamine to elderly patients.

In addition to age-related physiological changes, CYP2D6 genetic polymorphisms may further contribute to variability in fluvoxamine pharmacokinetics in elderly patients. CYP2D6 is a highly polymorphic enzyme, and genetic variation gives rise to distinct metabolizer phenotypes, including poor metabolizers (PMs), intermediate metabolizers (IMs), extensive metabolizers (EMs), and ultrarapid metabolizers (UMs). Ultrarapid metabolizers carry more than two copies of functional alleles, resulting in increased enzyme expression and enhanced metabolic clearance of CYP2D6 substrates. The PM phenotype is most commonly associated with nonfunctional alleles such as CYP2D6*3, CYP2D6*4, CYP2D6*5, and CYP2D6*6. Increased metabolic activity is typically observed in individuals carrying gene duplications or multiplication of wild-type alleles, including CYP2D6*1 and CYP2D6*2 [18]. The prevalence of the CYP2D6 PM phenotype varies across ethnic groups, occurring in approximately 5–10% of Caucasians, 1–2% of East Asians, and up to 7% of individuals of African descent [19].

Although the pharmacokinetic profile of fluvoxamine across CYP2D6 metabolizer phenotypes has been evaluated in healthy adults—where an approximately twofold increase in area under the concentration–time curve (AUC) was observed in PMs compared with EMs following single-dose administration [15]—the impact of CYP2D6 phenotype on fluvoxamine pharmacokinetics in elderly patients has not been systematically investigated. This knowledge gap is particularly relevant for elderly PMs, as reduced metabolic capacity associated with aging may further increase drug exposure in a population already predisposed to higher plasma concentrations. Consequently, CYP2D6 genetic variability may amplify inter-individual differences in fluvoxamine exposure and clinical response among older adults. Therefore, evaluating the combined effects of aging and CYP2D6 polymorphisms on fluvoxamine pharmacokinetics is essential to improve our understanding of the variability in drug response in the elderly and to inform potential individualized dosing strategies in high-risk patients.

In this study, the impact of CYP2D6 polymorphisms on fluvoxamine pharmacokinetics in elderly individuals was evaluated using a physiologically based pharmacokinetic (PBPK) modeling approach. Ultimately, this study aimed to inform clinically relevant, phenotype-guided dosing considerations for fluvoxamine in this vulnerable population.

## 2. Materials and Methods

### 2.1. PBPK Model Development

#### 2.1.1. Simcyp^®^ Setting for Population

The PBPK models were implemented within the Simcyp Simulator (version 25; Certara, Sheffield, UK). We utilized the Simcyp Healthy Volunteer population model for baseline population studies. To simulate geriatric pharmacokinetics, we used the default geriatrics population library file provided in Simcyp^®^. The physiological parameters incorporated into the geriatric model reflect age-related changes reported in the previous literature [20,21]. To simulate the impact of CYP2D6 phenotypes, we created entirely EM, IM, PM and UM populations by revising the default phenotype distribution to ensure uniform phenotype populations. Phenotype-specific CYP2D6 abundance values are provided in Supplementary Table S1. For trial design, we used a population size of 100, with 10 trials and 10 subjects per trial.

#### 2.1.2. Fluvoxamine Model

We used the fluvoxamine PBPK model within Simcyp version 25. The model input parameters are summarized in Supplementary Table S2. Briefly, the model incorporated the compound’s physicochemical characteristics, including molecular weight (318.3 g/mol), Log Po:w (3), and pKa (8.7). The fraction unbound in plasma (fup) was 0.14 and a blood-to-plasma ratio (B/P) was 1.5. Absorption was described using a first-order absorption model. Intestinal permeability inputs included an apparent MDCK II permeability of 31.7 × 10^−6^ cm/s (scalar 1.3983) and a predicted effective human intestinal permeability (Peff,man) of 5.67 × 10^−4^ cm/s. Distribution was represented using a minimal PBPK structure, where the volume of distribution at steady state (Vss) was 21 L/kg, inter-compartmental clearance (Q) was 0.5 L/h, and an adjusted peripheral compartment volume (Vsac) was 6 L/kg. Excretion was modeled using enzyme kinetic parameters to describe fluvoxamine metabolism via CYP2D6. The CYP2D6-mediated metabolic clearance was parameterized using a maximum metabolic rate (Vmax) of 70 pmol/min/pmol enzyme and a Michaelis–Menten constant (Km) of 38.6 μM.

### 2.2. PBPK Model Verification

To verify the fluvoxamine model, three single-dose studies and three multiple-dose studies of healthy adult or elderly populations were employed [9,22,23,24]. For the verification simulations, drug dose, administration schedule, age and sex distributions were matched to the design of the corresponding clinical studies. The prediction accuracy for AUC and maximum plasma concentration (Cmax) values was calculated as a ratio of mean observed to mean predicted values. Successful model performance was defined a priori by ratios of AUC and Cmax within a two-fold range of the mean observed values, as previously described [25,26].

To investigate whether the model replicates the observed impact of CYP2D6 polymorphisms on fluvoxamine PK in adults, where approximately a twofold increase in AUC was observed in PMs compared with EMs following single-dose administration [15], we simulated the single-dose PK of fluvoxamine in each phenotype by revising the default phenotype distribution to ensure uniform phenotype populations.

### 2.3. PBPK Model Application

The verified PBPK model was applied to predict (1) steady-state fluvoxamine pharmacokinetics in elderly individuals (65–98 years) and younger adults (18–65 years) and (2) the impact of CYP2D6 polymorphisms on fluvoxamine pharmacokinetics in the elderly population. Using the default healthy volunteers and geriatric population library file, fluvoxamine pharmacokinetics were simulated at daily doses of 50, 100, 150, and 200 mg in a non-stratified population. Further, to simulate the impact of CYP2D6 phenotypes, fluvoxamine pharmacokinetics were simulated in phenotype-specific populations consisting exclusively of EMs, IMs, PMs, or UMs. For daily doses exceeding 100 mg, the total daily dose was administered in two divided doses in accordance with the package insert. To evaluate appropriate dosing across CYP2D6 phenotype groups, the model-predicted steady-state trough concentrations were compared with the established therapeutic range of 60–230 ng/mL [27].

## 3. Results

### 3.1. PBPK Model Verification

The observed and simulated PK of fluvoxamine following single and multiple doses in healthy adults and elderly populations are summarized in Table 1. Overall, the predicted Cmax values were within 0.76 to 1.15 times the observed Cmax values, and the predicted AUC values ranged from 0.82 to 1.30 times the observed AUC values, falling within the acceptable two-fold limit.

Specifically, simulations of single-dose pharmacokinetics in elderly subjects (66–80 years) yielded predicted Cmax and AUC values within 0.79 to 1.14-fold of the observed data reported by Orlando et al. [9], supporting the applicability of the fluvoxamine PBPK model in elderly populations. Furthermore, according to the package insert, fluvoxamine exposure has been reported to be approximately two-fold higher in elderly patients compared with younger adults. This age-related increase in exposure was captured well by the model, with simulated exposure in elderly individuals being approximately 1.8-fold higher than that in younger adults when the same dosing regimen was administered.

Simulations were also conducted to evaluate the model’s ability to reproduce the observed impact of CYP2D6 polymorphisms on fluvoxamine pharmacokinetics. In a clinical single-dose study, fluvoxamine exposure in PMs was reported to be approximately twofold higher than in EMs [15]. Consistent with these observations, simulations following a 50 mg single dose predicted a 2.1-fold increase in AUC in PMs (1794.9 ng·h/mL) compared with EMs (854.8 ng·h/mL), demonstrating that the model accurately captures the influence of CYP2D6 phenotype on fluvoxamine pharmacokinetics.

### 3.2. Steady-State Fluvoxamine Pharmacokinetics in Older and Younger Adults

Because direct steady-state pharmacokinetic comparisons between elderly and younger adults are limited, the verified PBPK model was applied to simulate steady-state fluvoxamine pharmacokinetics in elderly individuals (65–98 years) and younger adults (18–65 years). The simulation results are summarized in Table 2.

At steady state, simulated Cmax values in the elderly population were approximately 1.6–1.7-fold higher than those in younger adults under identical dosing regimens. Similarly, AUC values were approximately 1.8-fold higher, and Cmin values were 1.8–2.0-fold higher in elderly individuals compared with younger adults. These findings suggest that dose titration strategies and maximum daily doses (up to 300 mg) commonly applied in younger adults may not be directly applicable to elderly patients and should be carefully reconsidered to ensure safety.

Fluvoxamine pharmacokinetics exhibited substantial inter-individual variability, with coefficients of variation for Cmin ranging from approximately 70% to 76% across dose levels. Further, variability was generally higher in older adults, highlighting the need for further evaluation of fluvoxamine pharmacokinetics in CYP2D6 phenotype-stratified populations to support precision dosing in the elderly.

### 3.3. Steady-State Fluvoxamine Pharmacokinetics in CYP2D6 Phenotype-Stratified Older Adults

The verified PBPK model was then applied to simulate steady-state fluvoxamine pharmacokinetics in CYP2D6 phenotype-stratified elderly individuals (65–98 years). Pharmacokinetic parameters were evaluated across different CYP2D6 phenotypes and dose levels. The results are summarized in Table 3.

At steady state, simulated AUC values in PMs were approximately 2.1-fold higher than those in EMs under identical dosing regimens, indicating a phenotype effect comparable in magnitude to that observed in younger adults. The IMs and UMs exhibited approximately 1.5-fold higher and 0.7-fold lower exposures, respectively, compared with EMs under the same dosing conditions.

**Figure 1:**
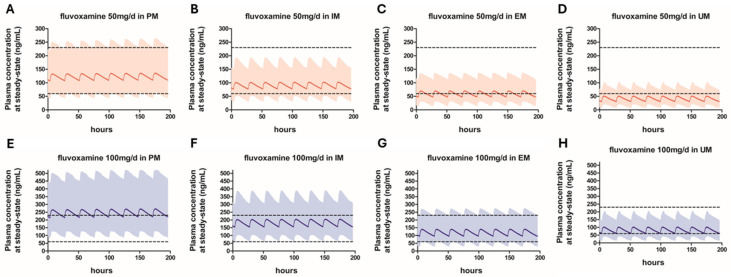
Predicted plasma concentration-time profiles of fluvoxamine 50 mg daily (**A**–**D**) and 100 mg daily (**E**–**H**) in ultrarapid metabolizer (UM), extensive metabolizer (EM), intermediate metabolizer (IM), and poor metabolizer (PM) elderly patients. The bold continuous lines represent the predicted mean concentrations, while the filled areas show the 5th to the 95th percentiles range. The broken horizontal lines represent the therapeutic index range (60–230 ng/mL).

The simulated steady-state plasma concentration–time profiles of fluvoxamine at 50 mg/day and 100 mg/day doses are shown in Figure 1. To explore potential strategies for fluvoxamine dose titration in elderly patients, simulated steady-state Cmin values were compared with the established therapeutic concentration range of 60–230 ng/mL.

At 50 mg/day, the mean Cmin values for PM and IM were above the lower therapeutic threshold (60 ng/mL), whereas the mean Cmin values for EM and UM were below this threshold, suggesting that this dose is likely suboptimal for these phenotypes. Overall, most PMs achieved concentrations within the therapeutic range at 50 mg/day, and further dose escalation may not be advisable due to the potential risk of excessive exposure.

At 100 mg/day, the mean Cmin in PMs was 219.4 ng/mL, approaching the upper limit of the therapeutic range (230 ng/mL), indicating that a substantial proportion of PMs may exceed the optimal exposure range at this dose. The mean Cmin value for IM was within the therapeutic range; however, the 95th percentiles exceeded the therapeutic threshold, suggesting that careful monitoring may be required when increasing the dose from 50 mg/day to 100 mg/day in this phenotype. In contrast, the mean Cmin for EM (95.5 ng/mL) was well within the therapeutic range, with most individuals achieving concentrations considered optimal. For UMs, the mean Cmin (61.8 ng/mL) was slightly above the lower therapeutic threshold. In UMs who fail to achieve an adequate clinical response at 100 mg/day, further dose escalation may be considered.

**Figure 2:**
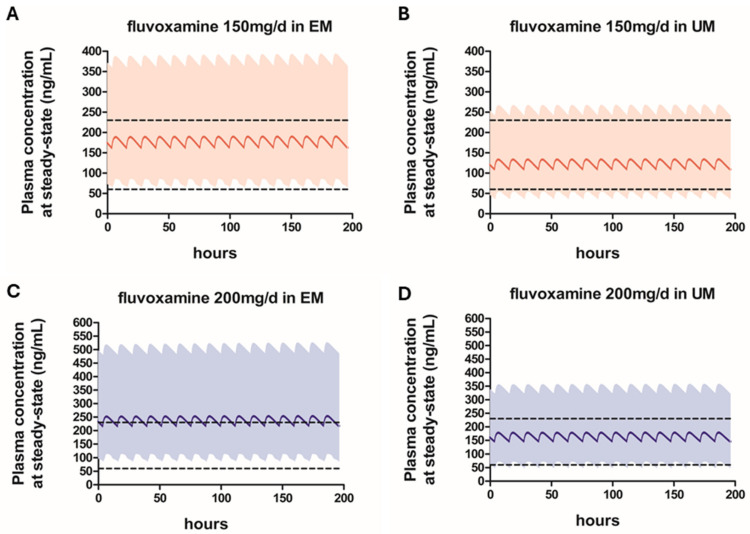
Predicted plasma concentration-time profiles of fluvoxamine 75 mg twice daily (**A**,**B**) and 100 mg twice daily (**C**,**D**) in ultrarapid metabolizer (UM) and extensive metabolizer (EM) elderly patients. The bold continuous lines represent the predicted mean concentrations, while the filled areas show the 5th to the 95th percentiles range. The broken horizontal lines represent therapeutic index range (60–230 ng/mL).

The simulated plasma concentration–time profiles for 75 mg twice daily and 100 mg twice daily (corresponding to total daily doses of 150 mg and 200 mg, respectively) in EMs and UMs are presented in Figure 2. At 150 mg/day, the mean Cmin values were 162.8 ng/mL for EMs and 109.3 ng/mL for UMs, both within the therapeutic range, with the majority of UMs achieving target concentrations. At 200 mg/day, the mean Cmin for EMs (217.3 ng/mL) approached the upper therapeutic threshold, indicating that a substantial proportion of EMs may be at increased risk of excessive exposure. In contrast, the mean Cmin for UMs remained within the therapeutic range, suggesting that, for UMs who do not achieve as adequate therapeutic response at 150 mg/day, dose escalation up to 200 mg/day may be considered with appropriate clinical monitoring.

## 4. Discussion

The findings of this study demonstrate that CYP2D6 genetic polymorphisms have a substantial impact on the pharmacokinetics of fluvoxamine in elderly patients, significantly contributing to the complexity and variability of drug exposure in this population. Using a PBPK modeling approach, we showed that elderly individuals experience markedly higher fluvoxamine exposure than younger adults, with this effect being most pronounced in PMs. These results indicate that standard dosing and titration strategies derived from younger adult populations may not be uniformly appropriate for elderly patients, particularly when genetic variability in drug metabolism is considered.

The fluvoxamine PBPK model applied in this study successfully reproduced observed pharmacokinetic profiles following both single and multiple dosing in healthy adults and elderly populations (Table 1). Although the fluvoxamine model implemented in Simcyp v25 has been used, detailed publications describing its development and validation—particularly with respect to elderly populations and CYP2D6 phenotype effects—remain limited. Therefore, we independently verified the model using available clinical pharmacokinetic data from younger and older adults, as well as phenotype-dependent exposure differences observed between CYP2D6 PMs and EMs. The model accurately captured the magnitude of CYP2D6-related exposure differences reported in adults, supporting its reliability for predicting fluvoxamine pharmacokinetics in elderly individuals stratified by CYP2D6 phenotype. As such, this verified PBPK model provides a valuable framework for predicting drug exposure and informing dose optimization in elderly patients, particularly in the context of genetic polymorphisms affecting drug metabolism.

To date, direct clinical comparisons of steady-state fluvoxamine pharmacokinetics between elderly and younger adults remain limited. To address this gap, we first applied the verified PBPK model to simulate steady-state pharmacokinetics in elderly individuals and younger adults. The simulations demonstrated that steady-state AUC values in elderly patients were approximately 1.8-fold higher than those in younger adults under identical dosing regimens (Table 2). This finding reinforces the notion that dose titration strategies and maximum daily doses (up to 300 mg/day) commonly applied in younger adults may not be directly transferable to elderly patients. Further, fluvoxamine pharmacokinetics in our simulations exhibited pronounced inter-individual variability, with coefficients of variation for steady-state Cmin ranging from approximately 70% to 76% across dose levels. Variability was generally greater in elderly individuals, highlighting the importance of accounting not only for mean exposure differences but also for the wide dispersion of concentrations within this population. These findings underscore the need for additional stratification by CYP2D6 phenotype to support more precise and individualized dosing strategies in elderly patients. These modeling results are consistent with clinical observations that elderly patients frequently exhibit greater inter-individual variability in antidepressant response and tolerability and are more susceptible to concentration-related adverse effects, such as sedation, dizziness, and cognitive impairment, which can increase the risk of treatment discontinuation [28,29,30,31]. In addition, clinically important adverse events such as SSRI-associated hyponatremia occur more frequently in older adults, underscoring the need for cautious dosing when systemic exposure is increased [32,33].

Phenotype-stratified simulations revealed that, at steady state, fluvoxamine AUC values in elderly PMs, IMs, and UMs were approximately 2.1-fold higher, 1.5-fold higher, and 0.7-fold lower, respectively, compared with EMs receiving the same dosing regimen. These results clearly indicate that distinct dose titration approaches are warranted across CYP2D6 phenotypes in elderly patients. At 50 mg/day, most PMs achieved therapeutic concentrations, suggesting that further dose escalation in this group may increase the risk of toxicity without additional benefit (Figure 1). In contrast, a substantial proportion of EMs and UMs remained below the therapeutic concentration range at this dose, indicating a risk of subtherapeutic exposure. For IMs, dose escalation beyond 50 mg/day may be considered in patients who fail to achieve an adequate clinical response; however, at 100 mg/day, the upper percentiles of Cmin exceeded the therapeutic threshold, suggesting that careful monitoring is required when increasing the dose in this phenotype. In EMs, administration of 100 mg/day resulted in therapeutic concentrations for the majority of individuals, whereas in UMs, the mean Cmin values were only slightly above the lower limit of the therapeutic range, indicating that additional dose increases may be necessary in some patients. At 150 mg/day, most UMs achieved target concentrations, and even at 200 mg/day, the mean Cmin values remained within the therapeutic range (Figure 2). Accordingly, for UMs who do not achieve an adequate clinical response at lower doses, dose escalation up to 200 mg/day may be considered, although this differs from the dosing approach in younger adults, for whom a maximum daily dose of 300 mg/day is permitted.

In our previous study of escitalopram, another SSRI commonly prescribed in older adults, PBPK simulations predicted that the escitalopram AUC was approximately 1.6-fold higher in elderly individuals compared with younger adults, consistent with an observed increase of approximately 1.5-fold [34]. In contrast, the impact of aging on fluvoxamine pharmacokinetics was more pronounced, with AUC values approximately 1.8-fold higher in simulations and 2.0-fold higher in observed clinical data in elderly individuals relative to younger adults. The greater age-related effect observed for fluvoxamine is likely attributable to its stronger dependence on hepatic metabolic capacity. While renal clearance is minimal for both agents, fluvoxamine undergoes extensive hepatic metabolism and exhibits a higher apparent oral clearance (approximately 80 L/h) than escitalopram (approximately 36 L/h) [35,36]. Consequently, age-related declines in hepatic metabolic function are more likely to result in larger increases in systemic exposure to fluvoxamine. With respect to genetic variability, the impact of CYP polymorphisms was comparable between the two drugs. PBPK simulations demonstrated that escitalopram exposure in CYP2C19 PMs was approximately 2.1-fold higher than in EMs, an effect similar in magnitude to the impact of CYP2D6 polymorphisms on fluvoxamine AUC observed in the present study. While escitalopram dose adjustment in elderly patients is relatively straightforward due to its narrow dosing range (10–20 mg/day), fluvoxamine exhibits a broader therapeutic dose range and greater pharmacokinetic sensitivity to aging, necessitating more refined, phenotype-specific dose titration.

Given that geriatric patients frequently experience polypharmacy, the potential for drug–drug interactions (DDIs) represents an important clinical consideration when evaluating fluvoxamine pharmacokinetics. In the present model, inhibition parameters (e.g., Ki values) were incorporated, enabling simulation of fluvoxamine as a perpetrator of CYP-mediated interactions. Additionally, fluvoxamine elimination was characterized using enzyme kinetic parameters describing CYP2D6-dependent metabolism, which allows for the evaluation of fluvoxamine as a victim drug when co-administered with CYP modulators. Therefore, the developed model provides a platform for predicting the impact of polypharmacy in geriatric populations and may support future investigations of clinically relevant DDIs.

The major strength of this study lies in its integrated evaluation of geriatric-specific physiological changes and CYP2D6 genetic variability using a PBPK modeling framework. By incorporating population-specific physiological alterations associated with aging, this approach enables a mechanistic assessment of CYP2D6-mediated variability in fluvoxamine pharmacokinetics in elderly patients—a topic that has been insufficiently addressed in prior studies focused primarily on younger adults.

Our study has several limitations. First, we utilized a default geriatric population model in Simcyp, which was constructed based on data from Caucasian geriatric populations. This may not fully capture ethnic differences in physiology or CYP2D6 allele frequency distributions. Second, the present study is simulation-based and lacks prospective clinical data to directly validate the proposed phenotype-specific dosing strategies. Future clinical studies in elderly patients are therefore warranted to confirm the safety and efficacy of these recommendations.

In conclusion, this study systematically evaluated the impact of CYP2D6 polymorphisms on fluvoxamine exposure in elderly patients and provides preliminary, phenotype-informed dosing considerations based on PBPK modeling. Our results suggest that doses of approximately 50 mg/day for PMs, 50–100 mg/day for IMs, 100 mg/day for EMs, and 150–200 mg/day for UMs may be appropriate in elderly patients, with cautious dose escalation and close monitoring in individuals who do not achieve adequate therapeutic response. These findings highlight the potential clinical value of CYP2D6 genotype-guided dosing of fluvoxamine in elderly populations, offering a pathway toward more individualized and safer antidepressant therapy. Further clinical investigations with larger cohorts are needed to validate these preliminary dosing recommendations and to establish their impact on clinical outcomes.

## Figures and Tables

**Table 1:** Comparison of Observed and Simulated Pharmacokinetic Parameters for Model Verification in Healthy Adult and Elderly Populations.

PK Study			PK Parameters			
			Observed		Simulated	
			Cmax (ng/mL)	AUC	Cmax (ng/mL)	AUC
Trial	Dose			(ng∙h/mL)		(ng∙h/mL)
Bahrami et al. [24] (24–30 years, 100% male)	100 mg Single Dose	Mean	46.2	1308 ^d^	45.8	1468
		SD	29.0	781	30.3	757
		Simulated/observed			0.99	1.12
Orlando et al. [9] (66–80 years, 100% male)	50 mg Single Dose	Mean	31	885 ^c^	24.4	1009
		SD	19	560	14.2	475
		Simulated/observed			0.79	1.14
Fleishaker et al. [23] (20–44 years, 100% male)	50 mg Single Dose	Mean	21.5	328.0 ^b^	22.9	405.6
		SD	4.9	84.6	15.1	217.8
		Simulated/observed			1.07	1.24
Fleishaker et al. [23] (20–44 years, 100% male)	100 mg daily	Mean	99.3	1762 ^b^	102.5	1988
		SD	35.0	737	55.7	1212
		Simulated/observed			1.03	1.13
Spigset et al. [22] (23–34 years, 100% male)	50 mg twice daily	Mean	77.7	761.1 ^a^	89.1	986.1
		SD	31.2	302.2	51.8	605.4
		Simulated/observed			1.15	1.30
Spigset et al. [22] (23–34 years, 100% male)	100 mg twice daily	Mean	234.9	2401.4 ^a^	178.3	1974.1
		SD	100.0	1031.1	103.6	1211.6
		Simulated/observed			0.76	0.82

PK, pharmacokinetic; SD, standard deviation; Cmax, maximum plasma concentration; AUC, area under the curve. ^a^ AUC (0–12 h); ^b^ AUC (0–24 h); ^c^ AUC (0–96 h); ^d^ AUC (0–∞).

**Table 2:** Simulated Pharmacokinetic Parameter Values for Fluvoxamine at Steady State in Older and Younger Adults.

Fluvoxamine Dose	Population	PK Parameters		
		Cmax	AUC *	Cmin
		(ng/mL)	(ng·h/mL)	(ng/mL)
50 mg/day	Older	89.2 ± 52.9	1881.7 ± 1236.3	66.0 ± 50.0
	Younger	55.8 ± 30.3	1073.1 ± 654.5	33.4 ± 25.4
100 mg/day	Older	178.6 ± 105.8	3767.0 ± 2474.0	132.2 ± 100.0
	Younger	111.7 ± 60.7	2147.4 ± 1309.3	66.8 ± 50.8
150 mg/day	Older	247.2 ± 155.9	2827.1 ± 1856.7	218.7 ± 152.8
	Younger	146.0 ± 84.2	1611.1 ± 982.4	118.1 ± 79.4
200 mg/day	Older	329.9 ± 208.0	3772.7 ± 2477.0	291.8 ± 203.8
	Younger	194.7 ± 112.4	2149.2 ± 1310.2	157.5 ± 105.9

Cmax, maximum plasma concentration; AUC, area under the curve; Cmin, minimum plasma concentration. Data are presented as mean ± standard deviation. * AUC (0–24 h) for 50 mg/day and 100 mg/day, AUC (0–12 h) for 150 and 200 mg/day.

**Table 3:** Simulated Pharmacokinetic Parameter Values for Fluvoxamine at Steady State in CYP2D6 Phenotype-Stratified Elderly Population.

Fluvoxamine Dose	CYP2D6 Phenotype	PK Parameters		
		Cmax	AUC *	Cmin
		(ng/mL)	(ng·h/mL)	(ng/mL)
50 mg/day	EM	70.2 ± 32.4	1429.5 ± 725.0	47.7 ± 28.7
	UM	51.0 ± 25.7	988.9 ± 554.7	30.8 ± 21.3
	IM	102.4 ± 43.3	2186.1 ± 996.6	77.7 ± 40.4
	PM	135.7 ± 57.5	2976.4 ± 1343.0	109.7 ± 54.8
100 mg/day	EM	140.6 ± 28.7	2863.4 ± 1453.0	95.5 ± 57.6
	UM	102.2 ± 51.4	1981.0 ± 1111.7	61.8 ± 42.8
	IM	205.1 ± 86.8	4376.9 ± 1996.4	155.6 ± 80.9
	PM	271.3 ± 115.0	5952.8 ± 2685.9	219.4 ± 109.7
150 mg/day	EM	190.5 ± 92.9	2149.5 ± 1091.7	162.8 ± 88.9
	UM	134.3 ± 71.7	1486.8 ± 835.1	109.3 ± 67.1
	IM	286.0 ± 126.5	3285.3 ± 1499.5	255.7 ± 123.2
	PM	384.7 ± 169.3	4464.6 ± 2014.4	353.0 ± 166.0
200 mg/day	EM	254.3 ± 124.1	2869.8 ± 1458.5	217.3 ± 118.7
	UM	179.3 ± 95.8	1985.0 ± 1115.6	145.9 ± 89.7
	IM	381.7 ± 169.0	4384.7 ± 2002.5	341.3 ± 164.5
	PM	521.9 ± 225.7	5952.8 ± 2685.9	470.6 ± 221.4

PK, pharmacokinetic; EM, extensive metabolizer; IM, intermediate metabolizer; PM, poor metabolizer; UM, ultra-rapid metabolizer; Cmax, maximum plasma concentration; AUC, area under the curve; Cmin, minimum plasma concentration. Data are presented as mean ± standard deviation. * AUC (0–24 h) for 50 mg/day and 100 mg/day, AUC (0–12 h) for 150 and 200 mg/day.

## Data Availability

The original contributions presented in this study are included in the article and Supplementary Materials. Further inquiries can be directed to the corresponding author.

## Supplementary Materials

The following supporting information can be downloaded at: https://www.mdpi.com/article/10.3390/pharmaceutics18020232/s1, Table S1: Hepatic CYP2D6 Enzyme Abundance Used in Simcyp® version 25 (Certara); Table S2: Parameters for the Fluvoxamine Model in Simcyp® version 25 (Certara).

## References

[1] Gildengers A., Houck P., Mulsant B., Pollock B., Mazumdar S., Miller M., Dew M., Frank E., Kupfer D., Reynolds C. (2002). Course and rate of antidepressant response in the very old. J. Affect. Disord..

[2] Kok R., Reynolds C. (2017). Management of Depression in Older Adults: A Review. JAMA.

[3] Sivertsen H., Bjorklof G.H., Engedal K., Selbaek G., Helvik A.S. (2015). Depression and Quality of Life in Older Persons: A Review. Dement. Geriatr. Cogn. Disord..

[4] Rhee T.G., Steffens D.C. (2020). Major depressive disorder and impaired health-related quality of life among US older adults. Int. J. Geriatr. Psychiatry.

[5] Draper B., Berman K. (2008). Tolerability of selective serotonin reuptake inhibitors: Issues relevant to the elderly. Drugs Aging.

[6] Omori I.M., Watanabe N., Nakagawa A., Cipriani A., Barbui C., McGuire H., Churchill R., Furukawa T.A. (2010). Fluvoxamine versus other anti-depressive agents for depression. Cochrane Database Syst. Rev..

[7] Hsu C.W., Tseng W.T., Wang L.J., Yang Y.H., Kao H.Y., Lin P.Y. (2022). Comparative effectiveness of antidepressants on geriatric depression: Real-world evidence from a population-based study. J. Affect. Disord..

[8] Rossini D., Serretti A., Franchini L., Mandelli L., Smeraldi E., De Ronchi D., Zanardi R. (2005). Sertraline versus fluvoxamine in the treatment of elderly patients with major depression: A double-blind, randomized trial. J. Clin. Psychopharmacol..

[9] Orlando R., De Martin S., Andrighetto L., Floreani M., Palatini P. (2010). Fluvoxamine pharmacokinetics in healthy elderly subjects and elderly patients with chronic heart failure. Br. J. Clin. Pharmacol..

[10] Drenth-van Maanen A.C., Wilting I., Jansen P.A.F. (2020). Prescribing medicines to older people-How to consider the impact of ageing on human organ and body functions. Br. J. Clin. Pharmacol..

[11] Kinirons M.T., O’Mahony M.S. (2004). Drug metabolism and ageing. Br. J. Clin. Pharmacol..

[12] Bayer A.J., Roberts N.A., Allen E.A., Horan M., Routledge P.A., Swift C.G., Byrne M.M., Clarkson A., Zussman B.D. (1989). The pharmacokinetics of paroxetine in the elderly. Acta Psychiatr. Scand. Suppl.

[13] Dawling S., Crome P., Braithwaite R. (1980). Pharmacokinetics of single oral doses of nortriptyline in depressed elderly hospital patients and young healthy volunteers. Clin. Pharmacokinet..

[14] Molden E., Waade R.B., Hoff M., Haslemo T. (2016). Impact of Ageing on Serum Concentrations of Risperidone and Its Active Metabolite in Patients with Known CYP2D6 Genotype. Basic Clin. Pharmacol. Toxicol..

[15] ANI Pharmaceuticals Fluvoxamine Maleate. Prescribing Information. https://www.anipharmaceuticals.com/products-detail.php?group=Fluvoxamine+Maleate+Tablets+USP&tablet=true.

[16] Filippatos T.D., Makri A., Elisaf M.S., Liamis G. (2017). Hyponatremia in the elderly: Challenges and solutions. Clin. Interv. Aging.

[17] Wagner W., Houser V., Wong L.F. (1996). The safety profile of fluvoxamine in elderly patients. Hum. Psychopharm. Clin..

[18] Zastrozhin M.S., Grishina E.A., Denisenko N.P., Skryabin V.Y., Markov D.D., Savchenko L.M., Bryun E.A., Sychev D.A. (2018). Effects of CYP2D6 genetic polymorphisms on the efficacy and safety of fluvoxamine in patients with depressive disorder and comorbid alcohol use disorder. Pharmgenomics Pers. Med..

[19] Bertilsson L., Dahl M.L., Dalen P., Al-Shurbaji A. (2002). Molecular genetics of CYP2D6: Clinical relevance with focus on psychotropic drugs. Br. J. Clin. Pharmacol..

[20] Schlender J.F., Meyer M., Thelen K., Krauss M., Willmann S., Eissing T., Jaehde U. (2016). Development of a Whole-Body Physiologically Based Pharmacokinetic Approach to Assess the Pharmacokinetics of Drugs in Elderly Individuals. Clin. Pharmacokinet.

[21] Chetty M., Johnson T.N., Polak S., Salem F., Doki K., Rostami-Hodjegan A. (2018). Physiologically based pharmacokinetic modelling to guide drug delivery in older people. Adv. Drug. Deliv. Rev..

[22] Spigset O., Granberg K., Hagg S., Soderstrom E., Dahlqvist R. (1998). Non-linear fluvoxamine disposition. Br. J. Clin Pharmacol..

[23] Fleishaker J.C., Hulst L.K. (1994). A pharmacokinetic and pharmacodynamic evaluation of the combined administration of alprazolam and fluvoxamine. Eur. J. Clin. Pharmacol..

[24] Bahrami G., Mohammadi B. (2007). Rapid and sensitive bioanalytical method for measurement of fluvoxamine in human serum using 4-chloro-7-nitrobenzofurazan as pre-column derivatization agent: Application to a human pharmacokinetic study. J. Chromatogr. B Analyt. Technol. Biomed Life Sci..

[25] Wang Y.H. (2010). Confidence assessment of the Simcyp time-based approach and a static mathematical model in predicting clinical drug-drug interactions for mechanism-based CYP3A inhibitors. Drug Metab. Dispos. Biol. Fate Chem..

[26] Galetin A., Ito K., Hallifax D., Houston J.B. (2005). CYP3A4 substrate selection and substitution in the prediction of potential drug-drug interactions. J. Pharmacol. Exp. Ther..

[27] Hiemke C., Bergemann N., Clement H., Conca A., Deckert J., Domschke K., Eckermann G., Egberts K., Gerlach M., Greiner C. (2018). Consensus Guidelines for Therapeutic Drug Monitoring in Neuropsychopharmacology: Update 2017. Pharmacopsychiatry.

[28] Sultana J., Spina E., Trifiro G. (2015). Antidepressant use in the elderly: The role of pharmacodynamics and pharmacokinetics in drug safety. Expert Opin. Drug Metab. Toxicol..

[29] Darowski A., Chambers S.A., Chambers D.J. (2009). Antidepressants and falls in the elderly. Drugs Aging.

[30] Alamo C., Lopez-Munoz F., Garcia-Garcia P., Garcia-Ramos S. (2014). Risk-benefit analysis of antidepressant drug treatment in the elderly. Psychogeriatrics.

[31] Chemali Z., Chahine L.M., Fricchione G. (2009). The use of selective serotonin reuptake inhibitors in elderly patients. Harv. Rev. Psychiatry.

[32] Kirby D., Ames D. (2001). Hyponatraemia and selective serotonin re-uptake inhibitors in elderly patients. Int. J. Geriatr. Psychiatry.

[33] Wee R., Lim W.K. (2004). Selective serotonin re-uptake inhibitors (SSRIs) and hyponatraemia in the elderly. Int. J. Geriatr. Psychiatry.

[34] Jang Y.J., Kim D.K., Lim S.W., Hong E. (2025). Impact of CYP2C19 Phenotype on Escitalopram Response in Geriatrics: Based on Physiologically-Based Pharmacokinetic Modeling and Clinical Observation. Clin. Pharmacol. Ther..

[35] DeVane C.L., Gill H.S. (1997). Clinical pharmacokinetics of fluvoxamine: Applications to dosage regimen design. J. Clin. Psychiatry.

[36] (2017). Lexapro. Prescribing Information. Allergan USA, Inc. https://www.accessdata.fda.gov/drugsatfda_docs/label/2017/021323s047lbl.pdf.

