# Supplementary material for: Evaluating the Impact of CYP2D6 Phenotype on Fluvoxamine Pharmacokinetics in Geriatric Patients Using Physiologically Based Pharmacokinetic Modeling
Source: Pharmaceutics. 2026 Feb 11;18(2):232. doi: 10.3390/pharmaceutics18020232 (PMC12944153; doi:10.3390/pharmaceutics18020232)
Supplement: Supplementary file 1 [file pharmaceutics-18-00232-s001.zip › Supplementary Table 2.pdf]

**Supplementary Table 2.** Parameters for the Fluvoxamine Model in Simcyp® version 25 (Certara).

| **Parameter** | **Value/Setting** |
|---|---|
| Physiochemical properties | |
| Molecular weight (g/mol) | 318.3 |
| Log $P_{o:w}$ | 3 |
| Compound type | Monoprotic Base |
| pKa | 8.7 |
| Polar surface area | 56.84 |
| Hydrogen bond donors | 1 |
| Hydrogen bond acceptors | 4 |
| B/P | 1.5 |
| $fu_p$ | 0.14 |
| Absorption | |
| Absorption model | First-order |
| MDCK II permeability (× $10^{-6}$ cm/s) | 31.7 (scalar: 1.3983) |
| $P_{eff,man}$ (× $10^{-4}$ cm/s) | 5.6745 |
| $k_a$ ($h^{-1}$) | 0.7 |
| $f_a$ | 1 |
| $f_{ugut}$ | 0.14 |
| $Q_{gut}$ (l/h) | 15.871 |
| Distribution | |
| Distribution model | Minimal PBPK model |
| $V_{ss}$ (l/kg) | 21 |
| Q (l/h) | 0.5 |

| | |
|---|---|
| $V_{sac}$ (l/kg) | 6 |
| Elimination | |
| CYP2D6 Vmax (pmol/min/pmol of isoform) | 70 |
| CYP2D6 Km (μM) | 38.6 |
| Active hepatic scalar | 3 |
| Additional clearance HLM (μl/min/mg protein) | 14 |
| Interaction | |
| CYP1A2 Ki (μM) | 0.002 |
| CYP2C9 Ki (μM) | 0.131 |
| CYP2C19 Ki (μM) | 0.0087 |
| CYP2D6 Ki (μM) | 0.403 |
| CYP3A4 Ki (μM) | 0.51 |
| CYP3A5 Ki (μM) | 0.51 |

B/P, blood-to-plasma ratio; fa, fraction available from dosage form; fugut, fraction unbound in enterocytes; fup, fraction unbound in plasma; ka, absorption rate constant; Ki, concentration of inhibitor that supports half maximal inhibition; Km, Michaelis constant; Log Po:w, logarithmic partition coefficient octonal:water; Peff,man, effective permeability in humans; pKa, logarithm of acid dissociation constant; Q, inter-compartment clearance; Qgut, flow rate for overall delivery of drug to the gut; Vmax, maximum metabolic rate; Vsac, single adjusted compartment volume; Vss, volume of distribution at steady state.
