# Supplementary material for: Evaluating the Impact of CYP2D6 Phenotype on Fluvoxamine Pharmacokinetics in Geriatric Patients Using Physiologically Based Pharmacokinetic Modeling
Source: Pharmaceutics. 2026 Feb 11;18(2):232. doi: 10.3390/pharmaceutics18020232 (PMC12944153; doi:10.3390/pharmaceutics18020232)
Supplement: Supplementary file 1 [file pharmaceutics-18-00232-s001.zip › Supplementary Table 1.pdf]

Supplementary Table 1. Hepatic CYP2D6 Enzyme Abundance Used in Simcyp® version 25 (Certara).

Hepatic Abundance (pmol/mg protein) (CV%)			
PMs	IMs	EMs	UMs
0	2.95 (65%)	9.4 (65%)	18.18 (65%)

EM, extensive metabolizer; IM, intermediate metabolizer; PM, poor metabolizer; UM, ultra-rapid metabolizer.
